# Supplementary material for: A Critical View of the Application of the APEX Software (Aqueous Photochemistry of Environmentally-Occurring Xenobiotics) to Predict Photoreaction Kinetics in Surface Freshwaters
Source: Molecules. 2019 Dec 18;25(1):9. doi: 10.3390/molecules25010009 (PMC7017383; doi:10.3390/molecules25010009)
Supplement: Supplementary file 1 [file molecules-25-00009-s001.zip › 3.2.4_gcc-4.4.0.zip!/3.2.4_gcc-4.4.0/3.2.4_gcc-4.4.0/doc/octave/html/interpreter/Applying-a-Function-to-an-Array.html]

Applying a Function to an Array - Untitled


Next: Special Utility Matrices,
Previous: Rearranging Matrices,
Up: Matrix Manipulation


---

### 16.3 Applying a Function to an Array

— Function File: **arrayfun** (func, a)  
— Function File: x = **arrayfun** (func, a)  
— Function File: x = **arrayfun** (func, a, b, ...)  
— Function File: [x, y, ...] = **arrayfun** (func, a, ...)  
— Function File: **arrayfun** (..., "UniformOutput", val)  
— Function File: **arrayfun** (..., "ErrorHandler", errfunc)  
> Execute a function on each element of an array. This is useful for
> functions that do not accept array arguments. If the function does
> accept array arguments it is better to call the function directly.
>
> The first input argument func can be a string, a function
> handle, an inline function or an anonymous function. The input
> argument a can be a logic array, a numeric array, a string
> array, a structure array or a cell array. By a call of the function
> arrayfun all elements of a are passed on to the named
> function func individually.
>
> The named function can also take more than two input arguments, with
> the input arguments given as third input argument b, fourth
> input argument c, ... If given more than one array input
> argument then all input arguments must have the same sizes, for
> example
>
> ```
>           arrayfun (@atan2, [1, 0], [0, 1])
>           => ans = [1.5708   0.0000]
> ```
>
> If the parameter val after a further string input argument
> "UniformOutput" is set `true` (the default), then the named
> function func must return a single element which then will be
> concatenated into the return value and is of type matrix. Otherwise,
> if that parameter is set to `false`, then the outputs are
> concatenated in a cell array. For example
>
> ```
>           arrayfun (@(x,y) x:y, "abc", "def", "UniformOutput", false)
>           => ans =
>           {
>             [1,1] = abcd
>             [1,2] = bcde
>             [1,3] = cdef
>           }
> ```
>
> If more than one output arguments are given then the named function
> must return the number of return values that also are expected, for
> example
>
> ```
>           [A, B, C] = arrayfun (@find, [10; 0], "UniformOutput", false)
>           =>
>           A =
>           {
>             [1,1] =  1
>             [2,1] = [](0x0)
>           }
>           B =
>           {
>             [1,1] =  1
>             [2,1] = [](0x0)
>           }
>           C =
>           {
>             [1,1] =  10
>             [2,1] = [](0x0)
>           }
> ```
>
> If the parameter errfunc after a further string input argument
> "ErrorHandler" is another string, a function handle, an inline
> function or an anonymous function, then errfunc defines a
> function to call in the case that func generates an error.
> The definition of the function must be of the form
>
> ```
>           function [...] = errfunc (s, ...)
> ```
>
> where there is an additional input argument to errfunc
> relative to func, given by s. This is a structure with
> the elements "identifier", "message" and "index", giving
> respectively the error identifier, the error message and the index of
> the array elements that caused the error. The size of the output
> argument of errfunc must have the same size as the output
> argument of func, otherwise a real error is thrown. For
> example
>
> ```
>           function y = ferr (s, x), y = "MyString"; endfunction
>           arrayfun (@str2num, [1234], \
>                     "UniformOutput", false, "ErrorHandler", @ferr)
>           => ans =
>           {
>            [1,1] = MyString
>           }
> ```
>
>
>
>
> **See also:** cellfun, spfun, structfun.

— Loadable Function: **bsxfun** (f, a, b)  
> Applies a binary function f element-wise to two matrix arguments
> a and b. The function f must be capable of accepting
> two column vector arguments of equal length, or one column vector
> argument and a scalar.
>
> The dimensions of a and b must be equal or singleton. The
> singleton dimensions of the matrices will be expanded to the same
> dimensionality as the other matrix.
>
>
>
> **See also:** arrayfun, cellfun.
